# Crystal structures of two deca­vanadates(V) with penta­aqua­manganese(II) pendant groups: (NMe_4_)_2_[V_10_O_28_{Mn(H_2_O)_5_}_2_]·5H_2_O and [NH_3_C(CH_2_OH)_3_]_2_[V_10_O_28_{Mn(H_2_O)_5_}_2_]·2H_2_O

**DOI:** 10.1107/S2056989014028230

**Published:** 2015-01-10

**Authors:** Maurício P. Franco, André Luis Rüdiger, Jaísa F. Soares, Giovana G. Nunes, David L. Hughes

**Affiliations:** a Departamento de Química, Universidade Federal do Paraná, Centro Politécnico, Jardim das Américas, 81530-900 Curitiba, PR, Brazil; b School of Chemistry, University of East Anglia, Norwich NR4 7TJ, England

**Keywords:** crystal structure, deca­vanadate, heteropolyanion, manganese(II), organic cations

## Abstract

Two heterometallic deca­vanadate(V) compounds, bis­(tetra­methyl­ammonium) deca­aquadi-μ_4_-oxido-tetra-μ_3_-oxido-hexa­deca-μ_2_-oxido-hexa­oxidodimang­anese(II)­deca­vanadate(V) penta­hydrate, (Me_4_N)_2_[V_10_O_28_{Mn(H_2_O)_5_}_2_]·5H_2_O, **A**, and bis­{[tris­(hy­droxy­meth­yl)meth­yl]ammonium} deca­aquadi-μ_4_-oxido-tetra-μ_3_-oxido-hexa­deca-μ_2_-oxido-hexa­oxidodimanganese(II)deca­vanadate(V) dihydrate, [NH_3_C(CH_2_OH)_3_]_2_[V_10_O_28_{Mn(H_2_O)_5_}_2_]·2H_2_O, **B**, have been synthesized under mild reaction conditions in an aqueous medium. Both polyanions present two [Mn(OH_2_)_5_]^2+^ complex units bound to the deca­vanadate cluster through oxide bridges. In **A**, the deca­vanadate unit has 2/*m* symmetry, whereas in **B** it has twofold symmetry. Apart from this, the main differences between **A** and **B** rest on the organic cations, tetra­methyl­ammonium and [tris­(hy­droxy­meth­yl)meth­yl]ammonium, respectively, and on the number and arrangement of the water mol­ecules of crystallization. In both compounds, the H atoms from the coordinating water mol­ecules participate in extensive three-dimensional hydrogen-bonding networks, which link the cluster units both directly and through solvent mol­ecules and, in **B**, through the ‘tris­’ cation hydroxyl groups. The cation in **B** also participates in N—H⋯O hydrogen bonds. A number of C—H⋯O inter­actions are also observed in both structures.

## Abstract 2

Two heterometallic deca­vanadate(V) hydrated salts with tetra­methyl­ammonium and [tris­(hy­droxy­meth­yl)meth­yl]ammonium counter-cations have been synthesized under mild reaction conditions in an aqueous medium. Both polyanions present two [Mn(OH_2_)_5_]^2+^ complex units bound to the deca­vanadate cluster through oxide bridges.

## Chemical context   

Research on the electronic properties, catalytic activities and biological roles of polyoxidovanadates has advanced enormously during the last few decades (Bošnjaković-Pavlović *et al.*, 2009 ▸; Liu & Zhou, 2010 ▸). Among these aggregates, the deca­vanadate(V) anion is the most intensively studied because of its biological effect on the activities of several enzymes (Aureliano & Ohlin, 2014 ▸) and its insulin-mimetic action (Chatkon *et al.*, 2013 ▸; Aureliano, 2014 ▸). The first functionalization of deca­vanadate anions, [H_n_V_10_O_28_]^(6−*n*)−^, with transition metal complexes was reported in 2007 (Li *et al.*, 2007 ▸). Since then, structures involving different binding modes with non-equivalent terminal and bridging oxido ligands have been described (Wang, Sun *et al.*, 2008 ▸; Wang, Yan *et al.*, 2008 ▸; Wang *et al.*, 2011 ▸; Long *et al.*, 2010 ▸; Xu *et al.*, 2012 ▸) and examples with first-row, *d*-block metal ions include complexation with copper(II), mangan­ese(II) and zinc(II) (Wang, Sun *et al.*, 2008 ▸; Wang *et al.*, 2011 ▸; Klištincová *et al.*, 2009 ▸, 2010 ▸; Pavliuk *et al.*, 2014 ▸).

Polyoxidovanadates containing manganese cations have been synthesized as ionic pairs (Shan & Huang, 1999 ▸; Lin *et al.*, 2011 ▸) or as heterometallic aggregates in which the oxidovanadate cluster acts as a metalloligand to the manganese complex (Inami *et al.*, 2009 ▸; Klištincová *et al.*, 2009 ▸). Recent inter­est in this kind of compound lies in a possible synergistic effect (involving the two metal elements) for the enhancement of the catalytic activity towards oxidation of organic substrates, such as in the photocatalytic degradation of dyes (Wu *et al.*, 2012 ▸).

While the synthesis of deca­vanadates with different organic cations as building blocks for supra­molecular assemblies is largely explored (da Silva *et al.*, 2003 ▸), a systematic procedure for their functionalization with transition metal complexes has not been well established. Our research group is currently involved in the synthesis of heterometallic polyoxidovanadates containing manganese(II) because of their potential activity as catalysts of olefin epoxidation. In this context, the reaction between NH_4_VO_3_ and mannitol to give **A** was carried out in aqueous solution in the presence of tetra­methyl­ammonium chloride (molar proportion 2:1:2), following a procedure described earlier by our group to produce the mixed-valence polyoxidovanadate (Me_4_N)_6_[V_15_O_36_(Cl)] (Nunes *et al.*, 2012 ▸). The dark-green solution obtained after reflux for 24 h received one molar equivalent of Mn(OAc)_2_·4H_2_O and was kept under reflux for 24 more hours. A mixture of dark-green crystals of (Me_4_N)_6_[V_15_O_36_(Cl)] and yellow prisms of (NMe_4_)_2_[V_10_O_28_{Mn(H_2_O)_5_}_2_]·5H_2_O (**A**) was isolated after four weeks at room temperature, the latter in 9% yield. Product **A** contains two tetra­methyl­ammonium cations and the [V_10_O_28_]^6–^ unit is covalently bound to two [Mn(OH_2_)_5_]^2+^ complexes by terminal oxido bridges.
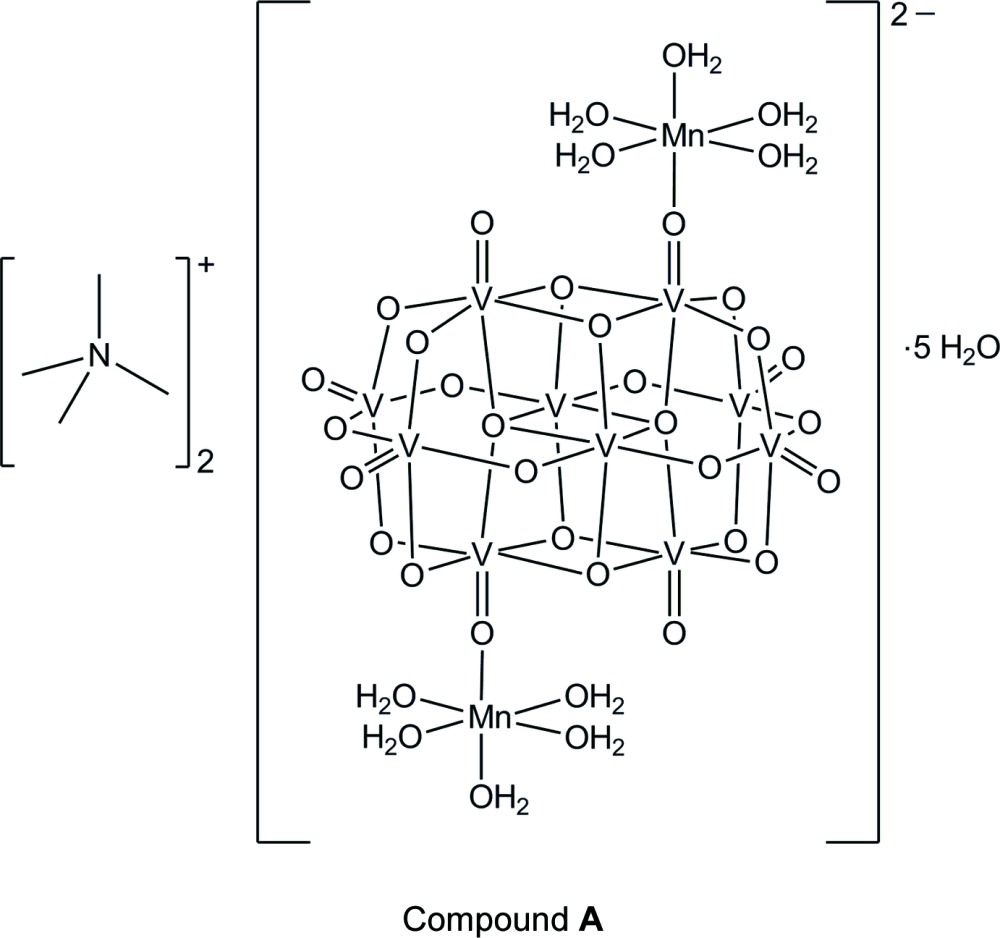


The rational synthesis of the heteropolyanion [V_10_O_28_{Mn(H_2_O)_5_}_2_]^2–^, in its turn, was achieved by reaction of NH_4_VO_3_ with tris­(hy­droxy­meth­yl)methyl­amine (‘tris­’) and manganese(II) chloride at pH 3 in a 5:3:1 molar proportion. Yellow crystals of [NH_3_C(CH_2_OH)_3_]_2_[V_10_O_28_{Mn(H_2_O)_5_}_2_]·2H_2_O (**B**) were isolated in 12% yield, as the only reaction product, after one week at room temperature. X-ray diffraction analyses revealed very similar structures for the heteropolyanions in **A** and **B**.
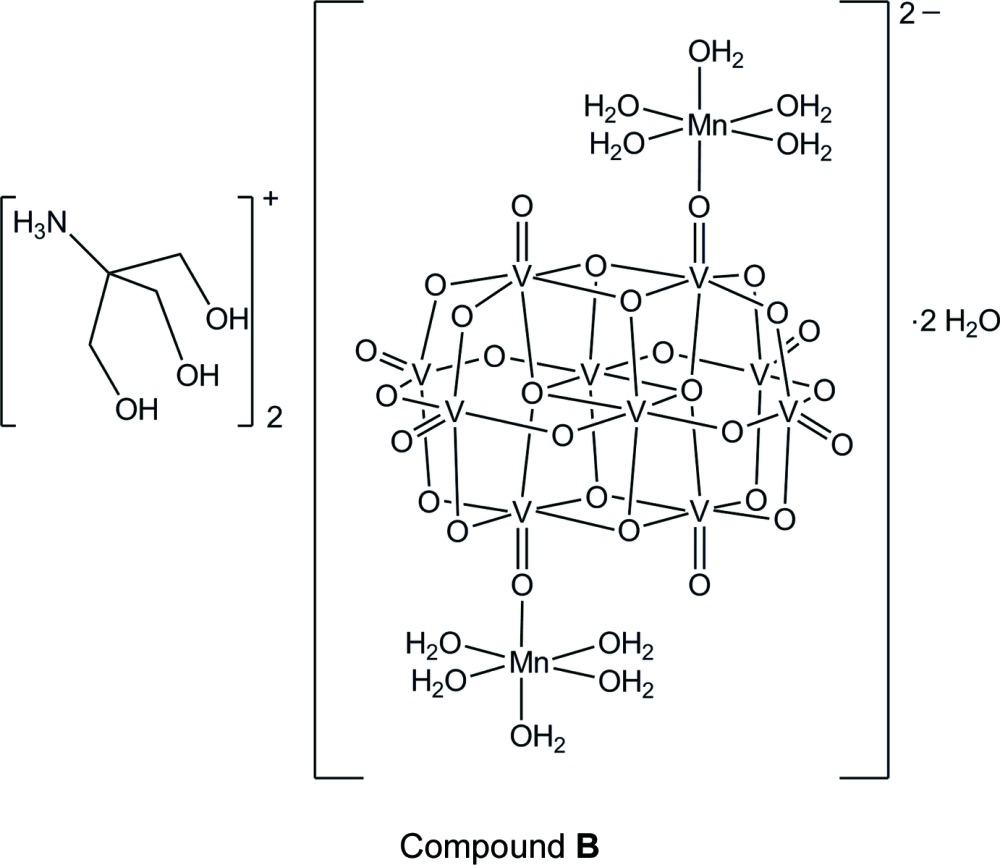


## Structural commentary   

**Figure 1:**
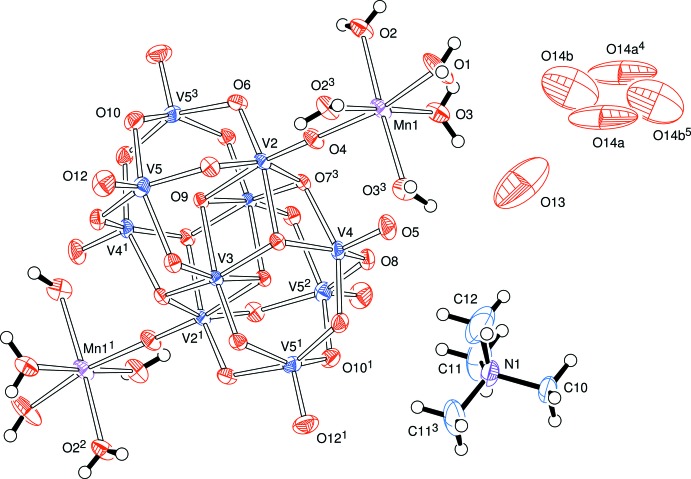
View of the components of (NMe_4_)_2_[V_10_O_28_{Mn(H_2_O)_5_}_2_]·5H_2_O, **A**, indicating the atom-numbering scheme. No H atoms were identified on the disordered solvent water mol­ecules. Displacement ellipsoids are drawn at the 50% probability level. [Symmetry codes: (1) 1 − *x*, *y*, 1 − *z*; (2) 1 − *x*, 1 − *y*, 1 − *z*; (3) *x*, 1 − *y*, *z*; (4) 1 − *x*, −*y*, −*z*; (5) 1 − *x*, *y*, −*z*.]

The anionic heteropolyanions are essentially identical in the two complexes. However, in **A**, the mol­ecule lies about the centre of the cell which is a point of 2/*m* symmetry, so that the unique part of the anionic cluster is one quarter of that heteropolyanion. The anion lies about a mirror plane which passes through the V2, V4 and manganese atoms, and there is a twofold symmetry axis which is perpendicular to the mirror plane and passes through V3 and the centre of the cell, Fig. 1 ▸.

**Figure 2:**
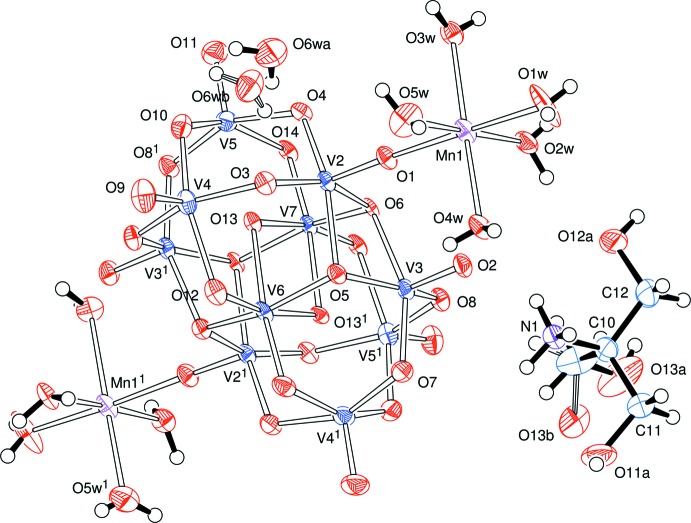
The corresponding view for [NH_3_C(CH_2_OH)_3_]_2_[V_10_O_28_{Mn(H_2_O)_5_}_2_]·2H_2_O, **B**. [Symmetry code: (1) 1 − *x*, *y*, 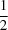 − *z*.]

**Figure 3:**
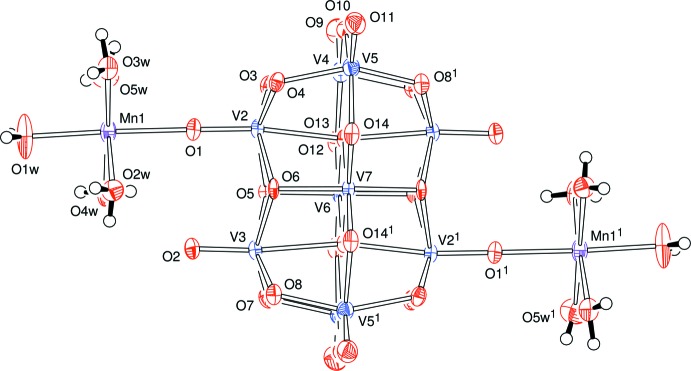
The anion of compound **B** viewed approximately down the *a* axis of the V_10_O_28_ moiety. [Symmetry code: (1) 1 − *x*, *y*, 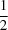 − *z*.]

The V_10_O_28_ moiety in the structure of compound **B** lies about a twofold symmetry axis which passes through the vanadium atoms V6 and V7, Fig. 2 ▸. This is the only crystallographic symmetry in this ion which, nevertheless, shows a very similar structure to that found in the ion in compound **A**; views showing this pseudo-symmetry are presented in Figs. 3 ▸, 4 ▸ and 5 ▸. The unique part here is one half of the anion. The previously reported analysis of this anion [with a 2-(2-hy­droxy­eth­yl)pyridinium cation] showed the cluster to be lying about an inversion centre (Klištincová *et al.*, 2009 ▸).

Bond angles and lengths determined for [V_10_O_28_{Mn(H_2_O)_5_}_2_]^2–^ are in the ranges reported in the literature (Klištincová *et al.*, 2009 ▸). In both our compounds, there is a wide range of V—O bond lengths. The vanadium atoms on the outer shell of the heteropolyanions, *e.g.* V4 and V5 in **A**, and V2–V5 in **B**, are five-coordinate with a square-pyramidal pattern; there is a sixth oxygen atom in the direction of an octa­hedral site but, at *ca* 2.3 Å from the vanadium atom, rather longer than the normal coordination distance. Of the five bonded oxygen atoms, the apical site (opposite the distant, sixth, site) has the shortest V—O distance, *ca* 1.6 Å, corres­ponding to a vanadyl group. The more ‘inter­nal’ vanadium atoms in each structure, *viz* V3 in **A**, and V6 and V7 in **B**, have more uniform V—O distances in more regular octa­hedral patterns.

## Supra­molecular features   

In both compounds, O—H⋯O hydrogen bonds from all the coordinating water mol­ecules link the anions with neighbouring anions, either directly, through both the cluster O atoms and the coordinating water mol­ecules, or indirectly through the solvent water mol­ecules (Tables 1 ▸ and 2 ▸). In compound **B**, additional hydroxyl groups are available in the ‘tris­’ cation, and these add further links in the extensive hydrogen bonding scheme. Additional C—H⋯O interactions are observed in the structures of both compounds.

## Database survey   

For structures with the [V_10_O_28_{Mn(H_2_O)_5_}_2_]^2–^ heteropolyanion, see: Klištincová *et al.* (2009 ▸). For structures with manganese(II) coordination complexes as counter-ions for [V_10_O_28_]^6–^, see: Klištincová *et al.* (2010 ▸); Shan & Huang (1999 ▸); Lin *et al.* (2011 ▸) and Mestiri *et al.* (2013 ▸).

## Synthesis and crystallization   

**General**

All reactions were performed in air with purified (Milli-Q^®^) water. Commercial reagents were used without purification. The starting materials NH_4_VO_3_, MnCl_2_·4H_2_O and Mn(OAc)_2_·4H_2_O were supplied by Aldrich, while mannitol [C_6_H_8_(OH)_6_] and (Me_4_N)Cl were purchased from USB and Merck, respectively. Infrared (FTIR) spectra were recorded on a BIORAD FTS-3500GX spectrophotometer from KBr pellets in the 400–4000 cm^−1^ region.

**Synthesis of (NMe_4_)_2_[V_10_O_28_{Mn(H_2_O)_5_}_2_]·5H_2_O (A)**

Solid NH_4_VO_3_ (0.500 g, 4.27 mmol) and [(CH_3_)_4_N]Cl (0.468 g, 4.27 mmol) were added to a solution of mannitol (0.366 g, 2.13 mmol) in 60 mL of water to produce a suspension that turned into a deep blue–greenish solution after one hour under reflux. After 24 more hours, a solution of Mn(OAc)_2_·4H_2_O (1.04 g, 4.27 mmol) in 10 mL of water was added to this reaction mixture, which remained under reflux for one more day. The solution was concentrated to one third of its initial volume and, after four weeks at room temperature, a mixture of deep-green crystals of (Me_4_N)_6_[V_15_O_36_(Cl)] (Nunes *et al.*, 2012 ▸) and yellow prisms of **A** was obtained, the latter in 9% yield based on vanadium (56 mg). The FTIR spectrum recorded for **A** shows the characteristic bands of the Me_4_N^+^ cation at 3031, 1639, 1485 and 1263 cm^−1^ and of the inorganic anion at 966, 833, 744, 584 and 455 cm^−1^.

**Synthesis of [NH_3_C(CH_2_OH)_3_]_2_[V_10_O_28_{Mn(H_2_O)_5_}_2_]·2H_2_O (B)**

A solution containing tris­(hy­droxy­meth­yl)methyl­amine (0.720 g, 6.0 mmol) in 20 mL of water was added to a solution of NH_4_VO_3_ (1.17 g, 10.0 mmol) in the same volume of solvent. This reaction mixture was then refluxed until it became a clear solution, after which its pH was adjusted to 3 with aqueous HCl. A solution of MnCl_2_·4H_2_O (0.394 g, 2.0 mmol) in 10 mL of water was then added as a layer on top of the reaction mixture and, after two weeks at room temperature, yellow crystals of **B** were obtained (180 mg) in 12% yield based on vanadium. The FTIR spectrum of **B** shows characteristic bands of the tris­H^+^ cation at 3188, 2927, 2856, 1743, 1637, 1417, 1161 and 1112 cm^−1^ and of the inorganic anion at 941, 842 and 684 cm^−1^.

## Refinement details   

Crystal data, data collection and structure refinement details for the two structures are summarized in Table 3 ▸.

Hydrogen atoms on the cation were included in idealized positions (with methyl and methyl­ene group C—H distances set at 0.96 and 0.97 Å, N—H at 0.89 Å and O—H at 0.82 Å) and their *U*_iso_ values were set to ride on the *U*_eq_ values of the parent atoms. Hydrogen atoms in the anions (on coordinating water mol­ecules) were located in difference maps and were refined freely.

There are two independent solvent water mol­ecules, one of which is disordered over two sites close to a centre of symmetry, in compound **A**. No hydrogen atoms were identified in these water mol­ecules.

In **B**, there is one solvent water mol­ecule which is disordered over two sites; the hydrogen atoms here were located in difference maps and were refined with distance restraints [O—H = 0.82 (2) Å].

## Supplementary Material

Crystal structure: contains datablock(s) Compound-A, Compound-B, global. DOI: 10.1107/S2056989014028230/hb7337sup1.cif

Structure factors: contains datablock(s) mpf-A. DOI: 10.1107/S2056989014028230/hb7337mpf-Asup2.hkl

Supporting information file. DOI: 10.1107/S2056989014028230/hb7337mpf-Asup4.cdx

Structure factors: contains datablock(s) mpf-B. DOI: 10.1107/S2056989014028230/hb7337mpf-Bsup3.hkl

Supporting information file. DOI: 10.1107/S2056989014028230/hb7337mpf-Bsup5.cdx

CCDC references: 1041495, 1041494

Additional supporting information:  crystallographic information; 3D view; checkCIF report

## Figures and Tables

**Figure 4:**
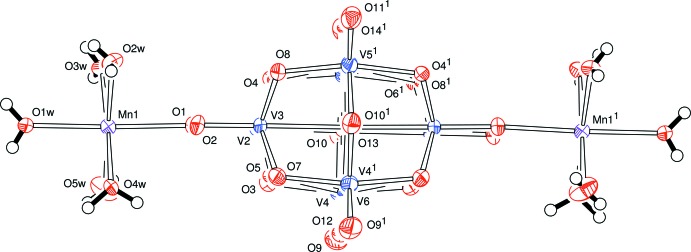
The anion of compound **B** viewed approximately down the *b* axis of the V_10_O_28_ moiety. [Symmetry code: (1) 1 − *x*, *y*, 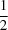 − *z*.]

**Figure 5:**
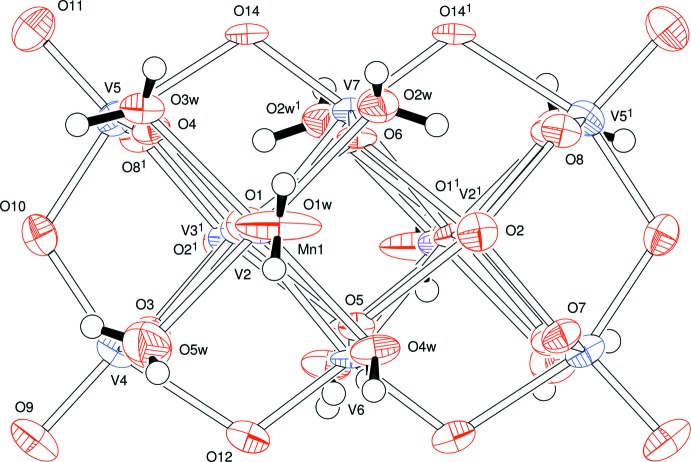
The anion of compound **B** viewed approximately down the *c* axis of the V_10_O_28_ moiety. [Symmetry code: (1) 1 − *x*, *y*, 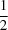 − *z*.]

**Table 1:** Hydrogen-bond geometry (Å, °) for compound **A** [Chem scheme1]

*D*—H⋯*A*	*D*—H	H⋯*A*	*D*⋯*A*	*D*—H⋯*A*
O1—H1*A*⋯O11^i^	0.76 (2)	1.97 (2)	2.7199 (14)	168 (2)
O2—H2*A*⋯O7^i^	0.72 (2)	2.04 (2)	2.7457 (15)	167 (2)
O2—H2*B*⋯O3^ii^	0.78 (2)	2.05 (2)	2.8295 (18)	178 (2)
O3—H3*A*⋯O6^ii^	0.74 (3)	1.92 (3)	2.6573 (16)	174 (3)
O3—H3*B*⋯O13	0.87 (3)	1.91 (3)	2.737 (3)	158 (3)
C10—H10*A*⋯O11^iii^	0.96	2.51	3.362 (2)	148
C10—H10*B*⋯O11^iv^	0.96	2.51	3.362 (2)	148
C10—H10*C*⋯O2^v^	0.96	2.58	3.370 (3)	139
C10—H10*C*⋯O2^vi^	0.96	2.57	3.370 (3)	141
C11—H11*A*⋯O8^vii^	0.96	2.48	3.384 (3)	156
C12—H12*A*⋯O12^iii^	0.96	2.60	3.474 (4)	152
C12—H12*C*⋯O12^iv^	0.96	2.59	3.474 (4)	153

Symmetry codes: (i) 
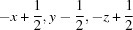
; (ii) 
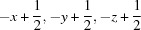
; (iii) 
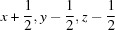
; (iv) 
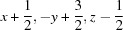
; (v) 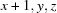; (vi) 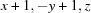; (vii) 
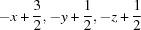
.

**Table 2:** Hydrogen-bond geometry (Å, °) for compound **B** [Chem scheme1]

*D*—H⋯*A*	*D*—H	H⋯*A*	*D*⋯*A*	*D*—H⋯*A*
O1*W*—H1*A*⋯O14^i^	0.70 (12)	2.01 (12)	2.703 (7)	167 (12)
O1*W*—H1*B*⋯O12^ii^	0.70 (7)	2.04 (8)	2.727 (8)	169 (8)
O2*W*—H2*A*⋯O5^iii^	0.60 (7)	2.12 (7)	2.716 (7)	172 (9)
O2*W*—H2*B*⋯O12*A*	0.87 (10)	2.01 (10)	2.858 (8)	164 (8)
O3*W*—H3*A*⋯O7^iii^	0.70 (9)	1.94 (9)	2.636 (7)	176 (10)
O3*W*—H3*B*⋯O11*A* ^iv^	0.88 (8)	1.91 (8)	2.752 (8)	160 (7)
O4*W*—H4*A*⋯O6^v^	0.82 (11)	1.90 (11)	2.708 (6)	167 (10)
O4*W*—H4*B*⋯O2*W* ^v^	0.76 (9)	2.12 (9)	2.871 (7)	169 (9)
O5*W*—H5*A*⋯O8^v^	0.55 (11)	2.18 (11)	2.725 (10)	170 (16)
O5*W*—H5*B*⋯O13*A* ^iv^	0.83 (14)	2.12 (13)	2.699 (12)	127 (12)
O5*W*—H5*B*⋯O13*B* ^iv^	0.83 (14)	1.95 (14)	2.77 (2)	168 (13)
N1—H1*C*⋯O3*W* ^v^	0.89	2.03	2.898 (7)	164
N1—H1*D*⋯O2	0.89	2.31	3.032 (7)	138
N1—H1*D*⋯O4*W*	0.89	2.45	3.105 (7)	130
N1—H1*E*⋯O4^v^	0.89	1.91	2.787 (6)	166
C11—H11*C*⋯O11^v^	0.97	2.46	3.392 (9)	160
O11*A*—H11*A*⋯O6*WA* ^v^	0.82	1.96	2.758 (12)	166
O12*A*—H12*A*⋯O3^iii^	0.82	1.94	2.756 (7)	174
C13—H13*E*⋯O2	0.97	2.40	3.280 (10)	151
O13*B*—H13*B*⋯O6*WB* ^vi^	0.77	1.92	2.60 (2)	148
O6*WA*—H6*A*⋯O3	0.82 (2)	2.23 (9)	2.966 (9)	150 (15)
O6*WA*—H6*B*⋯O10^vii^	0.82 (2)	2.16 (5)	2.952 (10)	165 (17)
O6*WB*—H6*C*⋯O3	0.82 (2)	2.03 (13)	2.802 (17)	156 (29)
O6*WB*—H6*D*⋯O10^vii^	0.82 (2)	1.93 (8)	2.720 (19)	161 (24)

Symmetry codes: (i) 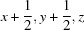; (ii) 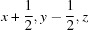; (iii) 
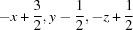
; (iv) 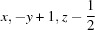; (v) 
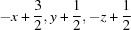
; (vi) 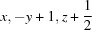; (vii) 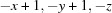.

**Table 3:** Experimental details

	Compound **A**	Compound **B**
Crystal data		
Chemical formula	(C_4_H_12_N)_2_·[Mn_2_V_10_O_28_(H_2_O)_10_]·5H_2_O	(C_4_H_12_NO_3_)_2_[Mn_2_V_10_O_28_(H_2_O)_10_]·2H_2_O
*M*_r_	1485.81	1527.76
Crystal system, space group	Monoclinic, *I*2/*m*	Monoclinic, *C*2/*c*
Temperature (K)	292	295
*a*, *b*, *c* (Å)	13.2434 (7), 9.6402 (5), 17.7628 (13)	19.3147 (8), 9.7733 (4), 22.7952 (10)
β (°)	98.626 (2)	96.392 (1)
*V* (Å^3^)	2242.1 (2)	4276.3 (3)
*Z*	2	4
Radiation type	Mo *K*α	Mo *K*α
μ (mm^−1^)	2.64	2.78
Crystal size (mm)	0.48 × 0.38 × 0.15	0.49 × 0.26 × 0.13

Data collection		
Diffractometer	Bruker D8 Venture/Photon 100 CMOS	Bruker D8 Venture/Photon 100 CMOS
Absorption correction	Multi-scan (*SADABS*; Bruker, 2012 ▸)	Multi-scan (*SADABS*; Bruker, 2012 ▸)
*T*_min_, *T*_max_	0.562, 0.746	0.542, 0.745
No. of measured, independent and observed [*I* > 2σ(*I*)] reflections	81983, 2953, 2752	71752, 3936, 3280
*R*_int_	0.025	0.039
(sin θ/λ)_max_ (Å^−1^)	0.668	0.605

Refinement		
*R*[*F* ^2^ > 2σ(*F* ^2^)], *wR*(*F* ^2^), *S*	0.020, 0.060, 1.09	0.052, 0.114, 1.12
No. of reflections	2953	3936
No. of parameters	194	385
No. of restraints	0	6
H-atom treatment	H atoms treated by a mixture of independent and constrained refinement	H atoms treated by a mixture of independent and constrained refinement
	*w* = 1/[σ^2^(*F*_o_^2^) + (0.0329*P*)^2^ + 2.2668*P*] where *P* = (*F*_o_^2^ + 2*F*_c_^2^)/3	*w* = 1/[σ^2^(*F*_o_^2^) + (0.0062*P*)^2^ + 110.7865*P*] where *P* = (*F*_o_^2^ + 2*F*_c_^2^)/3
Δρ_max_, Δρ_min_ (e Å^−3^)	0.58, −0.34	0.78, −1.11

Computer programs: *APEX2* and *SAINT* (Bruker, 2010 ▸), *SHELXS97*, *SHELXL97* (Sheldrick, 2008 ▸), *SHELXL2013* and *SHELXL2014* (Sheldrick, 2015 ▸), *ORTEP-3 for Windows* and *WinGX* (Farrugia, 2012 ▸).

## supplementary crystallographic information

### (Compound-A) Bis(tetramethylammonium) decaaquadi-µ4oxido-tetra-µ3-oxido-hexadeca-µ2-oxido-hexaoxidodimanganesedecavanadate pentahydrate Crystal data

**Table:**

(C_4_H_12_N)_2_·[Mn_2_V_10_O_28_(H_2_O)_10_]·5H_2_O	*F*(000) = 1480
*M**_r_* = 1485.81	*D*_x_ = 2.201 Mg m^−^^3^
Monoclinic, *I*2/*m*	Mo *K*α radiation, λ = 0.71073 Å
*a* = 13.2434 (7) Å	Cell parameters from 9256 reflections
*b* = 9.6402 (5) Å	θ = 3.1–28.3°
*c* = 17.7628 (13) Å	µ = 2.64 mm^−^^1^
β = 98.626 (2)°	*T* = 292 K
*V* = 2242.1 (2) Å^3^	Prism, yellow
*Z* = 2	0.48 × 0.38 × 0.15 mm

### (Compound-A) Bis(tetramethylammonium) decaaquadi-µ4oxido-tetra-µ3-oxido-hexadeca-µ2-oxido-hexaoxidodimanganesedecavanadate pentahydrate Data collection

**Table:**

Bruker D8 Venture/Photon 100 CMOS diffractometer	2953 independent reflections
Radiation source: fine-focus sealed tube	2752 reflections with *I* > 2σ(*I*)
Graphite monochromator	*R*_int_ = 0.025
Detector resolution: 10.4167 pixels mm^-1^	θ_max_ = 28.4°, θ_min_ = 3.1°
φ and ω scans	*h* = −17→17
Absorption correction: multi-scan (*SADABS*; Bruker, 2012)	*k* = −12→12
*T*_min_ = 0.562, *T*_max_ = 0.746	*l* = −23→23
81983 measured reflections	

### (Compound-A) Bis(tetramethylammonium) decaaquadi-µ4oxido-tetra-µ3-oxido-hexadeca-µ2-oxido-hexaoxidodimanganesedecavanadate pentahydrate Refinement

**Table:**

Refinement on *F*^2^	Primary atom site location: structure-invariant direct methods
Least-squares matrix: full	Secondary atom site location: difference Fourier map
*R*[*F*^2^ > 2σ(*F*^2^)] = 0.020	Hydrogen site location: mixed
*wR*(*F*^2^) = 0.060	H atoms treated by a mixture of independent and constrained refinement
*S* = 1.09	*w* = 1/[σ^2^(*F*_o_^2^) + (0.0329*P*)^2^ + 2.2668*P*] where *P* = (*F*_o_^2^ + 2*F*_c_^2^)/3
2953 reflections	(Δ/σ)_max_ = 0.001
194 parameters	Δρ_max_ = 0.58 e Å^−^^3^
0 restraints	Δρ_min_ = −0.34 e Å^−^^3^

### (Compound-A) Bis(tetramethylammonium) decaaquadi-µ4oxido-tetra-µ3-oxido-hexadeca-µ2-oxido-hexaoxidodimanganesedecavanadate pentahydrate Special details

**Table:**

Geometry. All e.s.d.'s (except the e.s.d. in the dihedral angle between two l.s. planes) are estimated using the full covariance matrix. The cell e.s.d.'s are taken into account individually in the estimation of e.s.d.'s in distances, angles and torsion angles; correlations between e.s.d.'s in cell parameters are only used when they are defined by crystal symmetry. An approximate (isotropic) treatment of cell e.s.d.'s is used for estimating e.s.d.'s involving l.s. planes.

### (Compound-A) Bis(tetramethylammonium) decaaquadi-µ4oxido-tetra-µ3-oxido-hexadeca-µ2-oxido-hexaoxidodimanganesedecavanadate pentahydrate Fractional atomic coordinates and isotropic or equivalent isotropic displacement parameters (Å^2^)

**Table:**

	*x*	*y*	*z*	*U*_iso_*/*U*_eq_	Occ. (<1)
Mn1	0.23106 (3)	0.5000	0.18528 (2)	0.02403 (8)	
V2	0.32741 (2)	0.5000	0.39643 (2)	0.01725 (8)	
V3	0.5000	0.66921 (3)	0.5000	0.01425 (8)	
V4	0.54714 (3)	0.5000	0.35620 (2)	0.01948 (8)	
V5	0.28084 (2)	0.65841 (2)	0.54017 (2)	0.02209 (7)	
O1	0.15020 (19)	0.5000	0.07177 (11)	0.0480 (6)	
O2	0.13101 (10)	0.32849 (12)	0.20645 (7)	0.0293 (2)	
O3	0.33574 (10)	0.33360 (12)	0.15931 (7)	0.0310 (3)	
O4	0.28574 (12)	0.5000	0.30500 (8)	0.0253 (3)	
O5	0.50366 (14)	0.5000	0.26708 (8)	0.0328 (4)	
O6	0.25845 (7)	0.36038 (10)	0.43170 (6)	0.0223 (2)	
O7	0.44883 (7)	0.62838 (10)	0.39540 (5)	0.01665 (18)	
O8	0.64321 (8)	0.36184 (11)	0.36540 (5)	0.0230 (2)	
O9	0.40458 (10)	0.5000	0.51485 (7)	0.0153 (2)	
O10	0.20825 (11)	0.5000	0.54989 (9)	0.0257 (3)	
O11	0.40317 (7)	0.77369 (10)	0.51675 (5)	0.02001 (19)	
O12	0.20299 (9)	0.78080 (13)	0.55243 (7)	0.0365 (3)	
H1A	0.1272 (17)	0.437 (2)	0.0491 (13)	0.053 (7)*	
H2A	0.1052 (16)	0.285 (2)	0.1767 (12)	0.038 (6)*	
H2B	0.1397 (15)	0.285 (2)	0.2440 (13)	0.038 (6)*	
H3A	0.3060 (19)	0.283 (3)	0.1339 (14)	0.053 (7)*	
H3B	0.392 (2)	0.359 (3)	0.1441 (17)	0.087 (10)*	
N1	0.85954 (17)	0.5000	0.23196 (11)	0.0348 (4)	
C10	0.9084 (2)	0.5000	0.16133 (14)	0.0373 (5)	
H10A	0.8883	0.4181	0.1321	0.056*	0.5
H10B	0.8870	0.5807	0.1315	0.056*	0.5
H10C	0.9813	0.5011	0.1750	0.056*	
C11	0.8902 (2)	0.3739 (2)	0.27733 (14)	0.0706 (8)	
H11A	0.8699	0.2930	0.2473	0.106*	
H11B	0.9630	0.3735	0.2920	0.106*	
H11C	0.8577	0.3735	0.3221	0.106*	
C12	0.7469 (3)	0.5000	0.2091 (2)	0.0895 (16)	
H12A	0.7269	0.4184	0.1797	0.134*	0.5
H12B	0.7142	0.5007	0.2539	0.134*	
H12C	0.7270	0.5810	0.1791	0.134*	0.5
O13	0.5022 (2)	0.3567 (5)	0.0856 (2)	0.1752 (17)	
O14A	0.5000	0.0973 (14)	0.0000	0.143 (14)	0.280 (18)
O14B	0.4760 (18)	0.0000	0.0562 (15)	0.158 (18)	0.220 (18)

### (Compound-A) Bis(tetramethylammonium) decaaquadi-µ4oxido-tetra-µ3-oxido-hexadeca-µ2-oxido-hexaoxidodimanganesedecavanadate pentahydrate Atomic displacement parameters (Å^2^)

**Table:**

	*U*^11^	*U*^22^	*U*^33^	*U*^12^	*U*^13^	*U*^23^
Mn1	0.03393 (17)	0.01631 (15)	0.01903 (15)	0.000	−0.00521 (12)	0.000
V2	0.01907 (15)	0.01704 (15)	0.01409 (15)	0.000	−0.00259 (11)	0.000
V3	0.01883 (15)	0.01043 (13)	0.01303 (14)	0.000	0.00088 (10)	0.000
V4	0.02432 (16)	0.02264 (17)	0.01183 (14)	0.000	0.00388 (11)	0.000
V5	0.02267 (12)	0.01995 (13)	0.02474 (13)	0.00444 (9)	0.00709 (9)	−0.00058 (9)
O1	0.0908 (17)	0.0167 (8)	0.0266 (9)	0.000	−0.0232 (9)	0.000
O2	0.0407 (6)	0.0190 (5)	0.0250 (6)	−0.0052 (4)	−0.0054 (5)	−0.0005 (4)
O3	0.0325 (6)	0.0240 (5)	0.0345 (6)	−0.0022 (5)	−0.0016 (5)	−0.0062 (5)
O4	0.0290 (7)	0.0270 (8)	0.0168 (6)	0.000	−0.0066 (5)	0.000
O5	0.0423 (9)	0.0413 (9)	0.0144 (7)	0.000	0.0030 (6)	0.000
O6	0.0219 (5)	0.0214 (5)	0.0223 (5)	−0.0047 (4)	−0.0010 (4)	−0.0013 (4)
O7	0.0214 (4)	0.0149 (4)	0.0128 (4)	0.0009 (3)	−0.0001 (3)	0.0016 (3)
O8	0.0285 (5)	0.0222 (5)	0.0195 (4)	0.0022 (4)	0.0078 (4)	−0.0029 (4)
O9	0.0191 (6)	0.0138 (6)	0.0128 (6)	0.000	0.0011 (5)	0.000
O10	0.0216 (7)	0.0268 (7)	0.0301 (8)	0.000	0.0082 (6)	0.000
O11	0.0256 (5)	0.0141 (4)	0.0202 (4)	0.0030 (4)	0.0031 (4)	−0.0001 (3)
O12	0.0341 (6)	0.0312 (6)	0.0458 (7)	0.0121 (5)	0.0113 (5)	−0.0029 (5)
N1	0.0484 (12)	0.0300 (10)	0.0304 (10)	0.000	0.0201 (9)	0.000
C10	0.0470 (14)	0.0381 (13)	0.0311 (12)	0.000	0.0204 (10)	0.000
C11	0.128 (2)	0.0406 (12)	0.0531 (13)	0.0137 (13)	0.0449 (15)	0.0168 (10)
C12	0.051 (2)	0.153 (5)	0.071 (3)	0.000	0.0329 (19)	0.000
O13	0.0805 (18)	0.238 (4)	0.220 (4)	0.022 (2)	0.064 (2)	0.065 (3)
O14A	0.027 (5)	0.059 (8)	0.33 (4)	0.000	−0.022 (10)	0.000
O14B	0.101 (15)	0.21 (4)	0.135 (19)	0.000	−0.077 (14)	0.000

### (Compound-A) Bis(tetramethylammonium) decaaquadi-µ4oxido-tetra-µ3-oxido-hexadeca-µ2-oxido-hexaoxidodimanganesedecavanadate pentahydrate Geometric parameters (Å, º)

**Table:**

Mn1—O1	2.1365 (18)	V5—O10	1.8264 (8)
Mn1—O4	2.1412 (14)	V5—O8^iii^	1.8322 (10)
Mn1—O2	2.1863 (12)	V5—O6^i^	1.9136 (10)
Mn1—O2^i^	2.1863 (12)	V5—O11	2.0579 (10)
Mn1—O3	2.2136 (12)	V5—O9	2.3326 (10)
Mn1—O3^i^	2.2136 (12)	V5—V5^i^	3.0543 (5)
V2—O4	1.6350 (14)	V5—V4^iii^	3.1068 (4)
V2—O6	1.7916 (10)	O1—H1A	0.76 (2)
V2—O6^i^	1.7916 (10)	O2—H2A	0.72 (2)
V2—O7^i^	2.0314 (10)	O2—H2B	0.78 (2)
V2—O7	2.0314 (10)	O3—H3A	0.74 (3)
V2—O9	2.1964 (12)	O3—H3B	0.87 (3)
V2—V4	3.0988 (5)	O6—V5^i^	1.9137 (10)
V2—V5	3.1152 (4)	O8—V5^iii^	1.8322 (10)
V2—V5^i^	3.1153 (4)	O9—V3^iii^	2.1041 (9)
V3—O11	1.6917 (10)	O9—V4^iii^	2.2840 (12)
V3—O11^ii^	1.6918 (10)	O9—V5^i^	2.3327 (10)
V3—O7	1.9205 (9)	O10—V5^i^	1.8264 (8)
V3—O7^ii^	1.9205 (9)	N1—C11^i^	1.481 (2)
V3—O9	2.1041 (9)	N1—C11	1.481 (2)
V3—O9^iii^	2.1041 (9)	N1—C12	1.486 (4)
V3—V5	3.0928 (3)	N1—C10	1.496 (3)
V3—V5^ii^	3.0928 (3)	C10—H10A	0.9600
V4—O5	1.6013 (15)	C10—H10B	0.9600
V4—O8^i^	1.8322 (10)	C10—H10C	0.9600
V4—O8	1.8322 (10)	C11—H11A	0.9600
V4—O7	1.9959 (10)	C11—H11B	0.9600
V4—O7^i^	1.9959 (10)	C11—H11C	0.9600
V4—O9^iii^	2.2840 (12)	C12—H12A	0.9600
V4—V5^ii^	3.1069 (4)	C12—H12B	0.9600
V4—V5^iii^	3.1069 (4)	C12—H12C	0.9600
V5—O12	1.6030 (11)		
			
O1—Mn1—O4	169.83 (9)	O5—V4—V5^iii^	135.13 (5)
O1—Mn1—O2	86.07 (6)	O8^i^—V4—V5^iii^	82.35 (3)
O4—Mn1—O2	87.28 (4)	O8—V4—V5^iii^	32.02 (3)
O1—Mn1—O2^i^	86.07 (6)	O7—V4—V5^iii^	123.83 (3)
O4—Mn1—O2^i^	87.28 (4)	O7^i^—V4—V5^iii^	86.97 (3)
O2—Mn1—O2^i^	98.27 (7)	O9^iii^—V4—V5^iii^	48.37 (3)
O1—Mn1—O3	92.53 (6)	V2—V4—V5^iii^	119.596 (11)
O4—Mn1—O3	94.47 (4)	V5^ii^—V4—V5^iii^	58.884 (11)
O2—Mn1—O3	84.40 (5)	O12—V5—O10	104.13 (6)
O2^i^—Mn1—O3	176.89 (5)	O12—V5—O8^iii^	103.30 (6)
O1—Mn1—O3^i^	92.53 (6)	O10—V5—O8^iii^	92.80 (6)
O4—Mn1—O3^i^	94.47 (4)	O12—V5—O6^i^	101.58 (6)
O2—Mn1—O3^i^	176.88 (5)	O10—V5—O6^i^	90.67 (6)
O2^i^—Mn1—O3^i^	84.40 (5)	O8^iii^—V5—O6^i^	153.19 (5)
O3—Mn1—O3^i^	92.89 (7)	O12—V5—O11	99.90 (6)
O4—V2—O6	103.51 (5)	O10—V5—O11	155.80 (5)
O4—V2—O6^i^	103.51 (5)	O8^iii^—V5—O11	84.37 (4)
O6—V2—O6^i^	97.40 (7)	O6^i^—V5—O11	81.68 (4)
O4—V2—O7^i^	98.09 (5)	O12—V5—O9	173.12 (5)
O6—V2—O7^i^	89.49 (4)	O10—V5—O9	82.29 (4)
O6^i^—V2—O7^i^	155.03 (4)	O8^iii^—V5—O9	78.56 (4)
O4—V2—O7	98.09 (5)	O6^i^—V5—O9	75.59 (4)
O6—V2—O7	155.03 (4)	O11—V5—O9	73.59 (3)
O6^i^—V2—O7	89.49 (4)	O12—V5—V5^i^	137.39 (5)
O7^i^—V2—O7	75.07 (5)	O10—V5—V5^i^	33.27 (4)
O4—V2—O9	172.10 (7)	O8^iii^—V5—V5^i^	83.88 (3)
O6—V2—O9	81.57 (4)	O6^i^—V5—V5^i^	84.57 (3)
O6^i^—V2—O9	81.57 (4)	O11—V5—V5^i^	122.69 (3)
O7^i^—V2—O9	75.72 (4)	O9—V5—V5^i^	49.10 (2)
O7—V2—O9	75.72 (4)	O12—V5—V3	130.66 (5)
O4—V2—V4	87.69 (6)	O10—V5—V3	125.13 (4)
O6—V2—V4	128.77 (3)	O8^iii^—V5—V3	79.01 (3)
O6^i^—V2—V4	128.77 (3)	O6^i^—V5—V3	77.29 (3)
O7^i^—V2—V4	39.28 (3)	O11—V5—V3	30.76 (3)
O7—V2—V4	39.28 (3)	O9—V5—V3	42.84 (2)
O9—V2—V4	84.41 (4)	V5^i^—V5—V3	91.929 (7)
O4—V2—V5	137.34 (4)	O12—V5—V4^iii^	135.29 (5)
O6—V2—V5	84.70 (3)	O10—V5—V4^iii^	83.26 (4)
O6^i^—V2—V5	34.01 (3)	O8^iii^—V5—V4^iii^	32.02 (3)
O7^i^—V2—V5	124.08 (3)	O6^i^—V5—V4^iii^	122.63 (3)
O7—V2—V5	87.88 (3)	O11—V5—V4^iii^	81.71 (3)
O9—V2—V5	48.39 (3)	O9—V5—V4^iii^	47.04 (3)
V4—V2—V5	119.816 (10)	V5^i^—V5—V4^iii^	60.558 (6)
O4—V2—V5^i^	137.34 (4)	V3—V5—V4^iii^	61.520 (9)
O6—V2—V5^i^	34.02 (3)	O12—V5—V2	133.04 (5)
O6^i^—V2—V5^i^	84.70 (3)	O10—V5—V2	80.75 (4)
O7^i^—V2—V5^i^	87.88 (3)	O8^iii^—V5—V2	123.30 (3)
O7—V2—V5^i^	124.08 (3)	O6^i^—V5—V2	31.58 (3)
O9—V2—V5^i^	48.39 (3)	O11—V5—V2	80.81 (3)
V4—V2—V5^i^	119.816 (10)	O9—V5—V2	44.75 (3)
V5—V2—V5^i^	58.710 (11)	V5^i^—V5—V2	60.645 (6)
O11—V3—O11^ii^	106.92 (7)	V3—V5—V2	61.274 (8)
O11—V3—O7	97.20 (4)	V4^iii^—V5—V2	91.545 (10)
O11^ii^—V3—O7	96.82 (4)	Mn1—O1—H1A	127.0 (17)
O11—V3—O7^ii^	96.82 (4)	Mn1—O2—H2A	123.2 (17)
O11^ii^—V3—O7^ii^	97.20 (4)	Mn1—O2—H2B	122.5 (15)
O7—V3—O7^ii^	156.35 (6)	H2A—O2—H2B	108 (2)
O11—V3—O9	87.37 (4)	Mn1—O3—H3A	108.1 (19)
O11^ii^—V3—O9	165.69 (5)	Mn1—O3—H3B	117 (2)
O7—V3—O9	80.27 (4)	H3A—O3—H3B	114 (3)
O7^ii^—V3—O9	81.45 (4)	V2—O4—Mn1	179.96 (10)
O11—V3—O9^iii^	165.69 (5)	V2—O6—V5^i^	114.40 (5)
O11^ii^—V3—O9^iii^	87.38 (4)	V3—O7—V4	108.10 (4)
O7—V3—O9^iii^	81.45 (4)	V3—O7—V2	106.33 (4)
O7^ii^—V3—O9^iii^	80.27 (4)	V4—O7—V2	100.60 (4)
O9—V3—O9^iii^	78.34 (6)	V5^iii^—O8—V4	115.96 (5)
O11—V3—V5	38.47 (3)	V3^iii^—O9—V3	101.66 (6)
O11^ii^—V3—V5	145.38 (4)	V3^iii^—O9—V2	94.70 (4)
O7—V3—V5	90.52 (3)	V3—O9—V2	94.70 (4)
O7^ii^—V3—V5	88.69 (3)	V3^iii^—O9—V4^iii^	92.45 (4)
O9—V3—V5	48.93 (3)	V3—O9—V4^iii^	92.45 (4)
O9^iii^—V3—V5	127.22 (3)	V2—O9—V4^iii^	168.68 (7)
O11—V3—V5^ii^	145.38 (4)	V3^iii^—O9—V5	169.81 (5)
O11^ii^—V3—V5^ii^	38.47 (3)	V3—O9—V5	88.231 (11)
O7—V3—V5^ii^	88.68 (3)	V2—O9—V5	86.86 (4)
O7^ii^—V3—V5^ii^	90.52 (3)	V4^iii^—O9—V5	84.59 (4)
O9—V3—V5^ii^	127.22 (3)	V3^iii^—O9—V5^i^	88.232 (11)
O9^iii^—V3—V5^ii^	48.93 (3)	V3—O9—V5^i^	169.81 (5)
V5—V3—V5^ii^	176.145 (13)	V2—O9—V5^i^	86.86 (4)
O5—V4—O8^i^	103.31 (5)	V4^iii^—O9—V5^i^	84.59 (4)
O5—V4—O8	103.30 (5)	V5—O9—V5^i^	81.79 (4)
O8^i^—V4—O8	93.26 (7)	V5^i^—O10—V5	113.47 (8)
O5—V4—O7	100.85 (6)	V3—O11—V5	110.76 (5)
O8^i^—V4—O7	89.92 (4)	C11^i^—N1—C11	110.3 (3)
O8—V4—O7	154.18 (4)	C11^i^—N1—C12	109.32 (18)
O5—V4—O7^i^	100.85 (6)	C11—N1—C12	109.32 (18)
O8^i^—V4—O7^i^	154.18 (4)	C11^i^—N1—C10	109.79 (14)
O8—V4—O7^i^	89.92 (4)	C11—N1—C10	109.79 (14)
O7—V4—O7^i^	76.64 (6)	C12—N1—C10	108.3 (2)
O5—V4—O9^iii^	175.24 (8)	N1—C10—H10A	109.5
O8^i^—V4—O9^iii^	79.88 (4)	N1—C10—H10B	109.5
O8—V4—O9^iii^	79.88 (4)	H10A—C10—H10B	109.5
O7—V4—O9^iii^	75.47 (4)	N1—C10—H10C	109.5
O7^i^—V4—O9^iii^	75.48 (4)	H10A—C10—H10C	109.5
O5—V4—V2	90.98 (7)	H10B—C10—H10C	109.5
O8^i^—V4—V2	130.01 (3)	N1—C11—H11A	109.5
O8—V4—V2	130.01 (3)	N1—C11—H11B	109.5
O7—V4—V2	40.12 (3)	H11A—C11—H11B	109.5
O7^i^—V4—V2	40.12 (3)	N1—C11—H11C	109.5
O9^iii^—V4—V2	84.26 (3)	H11A—C11—H11C	109.5
O5—V4—V5^ii^	135.13 (5)	H11B—C11—H11C	109.5
O8^i^—V4—V5^ii^	32.02 (3)	N1—C12—H12A	109.5
O8—V4—V5^ii^	82.35 (3)	N1—C12—H12B	109.5
O7—V4—V5^ii^	86.97 (3)	H12A—C12—H12B	109.5
O7^i^—V4—V5^ii^	123.83 (3)	N1—C12—H12C	109.5
O9^iii^—V4—V5^ii^	48.37 (3)	H12A—C12—H12C	109.5
V2—V4—V5^ii^	119.596 (11)	H12B—C12—H12C	109.5
			
O4—V2—O6—V5^i^	−174.84 (6)	O12—V5—O10—V5^i^	−178.86 (8)
O6^i^—V2—O6—V5^i^	−68.97 (7)	O8^iii^—V5—O10—V5^i^	−74.39 (8)
O7^i^—V2—O6—V5^i^	86.96 (6)	O6^i^—V5—O10—V5^i^	79.02 (8)
O7—V2—O6—V5^i^	35.96 (13)	O11—V5—O10—V5^i^	8.1 (2)
O9—V2—O6—V5^i^	11.32 (5)	O9—V5—O10—V5^i^	3.67 (8)
V4—V2—O6—V5^i^	87.13 (6)	V3—V5—O10—V5^i^	4.14 (11)
V5—V2—O6—V5^i^	−37.34 (5)	V4^iii^—V5—O10—V5^i^	−43.77 (7)
O5—V4—O8—V5^iii^	174.45 (7)	V2—V5—O10—V5^i^	48.91 (7)
O8^i^—V4—O8—V5^iii^	69.96 (7)	O11^ii^—V3—O11—V5	−178.88 (6)
O7—V4—O8—V5^iii^	−26.63 (13)	O7—V3—O11—V5	81.71 (5)
O7^i^—V4—O8—V5^iii^	−84.41 (6)	O7^ii^—V3—O11—V5	−79.19 (5)
O9^iii^—V4—O8—V5^iii^	−9.15 (5)	O9—V3—O11—V5	1.87 (5)
V2—V4—O8—V5^iii^	−82.75 (6)	O9^iii^—V3—O11—V5	−1.9 (2)
V5^ii^—V4—O8—V5^iii^	39.78 (5)	V5^ii^—V3—O11—V5	179.893 (6)

Symmetry codes: (i) *x*, −*y*+1, *z*; (ii) −*x*+1, *y*, −*z*+1; (iii) −*x*+1, −*y*+1, −*z*+1.

### (Compound-A) Bis(tetramethylammonium) decaaquadi-µ4oxido-tetra-µ3-oxido-hexadeca-µ2-oxido-hexaoxidodimanganesedecavanadate pentahydrate Hydrogen-bond geometry (Å, º)

**Table:**

*D*—H···*A*	*D*—H	H···*A*	*D*···*A*	*D*—H···*A*
O1—H1*A*···O11^iv^	0.76 (2)	1.97 (2)	2.7199 (14)	168 (2)
O2—H2*A*···O7^iv^	0.72 (2)	2.04 (2)	2.7457 (15)	167 (2)
O2—H2*B*···O3^v^	0.78 (2)	2.05 (2)	2.8295 (18)	178 (2)
O3—H3*A*···O6^v^	0.74 (3)	1.92 (3)	2.6573 (16)	174 (3)
O3—H3*B*···O13	0.87 (3)	1.91 (3)	2.737 (3)	158 (3)
C10—H10*A*···O11^vi^	0.96	2.51	3.362 (2)	148
C10—H10*B*···O11^vii^	0.96	2.51	3.362 (2)	148
C10—H10*C*···O2^viii^	0.96	2.58	3.370 (3)	139
C10—H10*C*···O2^ix^	0.96	2.57	3.370 (3)	141
C11—H11*A*···O8^x^	0.96	2.48	3.384 (3)	156
C12—H12*A*···O12^vi^	0.96	2.60	3.474 (4)	152
C12—H12*C*···O12^vii^	0.96	2.59	3.474 (4)	153

Symmetry codes: (iv) −*x*+1/2, *y*−1/2, −*z*+1/2; (v) −*x*+1/2, −*y*+1/2, −*z*+1/2; (vi) *x*+1/2, *y*−1/2, *z*−1/2; (vii) *x*+1/2, −*y*+3/2, *z*−1/2; (viii) *x*+1, *y*, *z*; (ix) *x*+1, −*y*+1, *z*; (x) −*x*+3/2, −*y*+1/2, −*z*+1/2.

### (Compound-B) Bis{[tris(hydroxymethyl)methyl]ammonium} decaaquadi-µ4oxido-tetra-µ3-oxido-hexadeca-µ2-oxido-hexaoxidodimanganesedecavanadate dihydrate Crystal data

**Table:**

(C_4_H_12_NO_3_)_2_[Mn_2_V_10_O_28_(H_2_O)_10_]·2H_2_O	*F*(000) = 3032
*M**_r_* = 1527.76	*D*_x_ = 2.373 Mg m^−^^3^
Monoclinic, *C*2/*c*	Mo *K*α radiation, λ = 0.71073 Å
*a* = 19.3147 (8) Å	Cell parameters from 38099 reflections
*b* = 9.7733 (4) Å	θ = 3.0–25.4°
*c* = 22.7952 (10) Å	µ = 2.78 mm^−^^1^
β = 96.392 (1)°	*T* = 295 K
*V* = 4276.3 (3) Å^3^	Plate, yellow
*Z* = 4	0.49 × 0.26 × 0.13 mm

### (Compound-B) Bis{[tris(hydroxymethyl)methyl]ammonium} decaaquadi-µ4oxido-tetra-µ3-oxido-hexadeca-µ2-oxido-hexaoxidodimanganesedecavanadate dihydrate Data collection

**Table:**

Bruker D8 Venture/Photon 100 CMOS diffractometer	3936 independent reflections
Radiation source: fine-focus sealed tube	3280 reflections with *I* > 2σ(*I*)
Graphite monochromator	*R*_int_ = 0.039
Detector resolution: 10.4167 pixels mm^-1^	θ_max_ = 25.5°, θ_min_ = 2.9°
φ and ω scans	*h* = −23→23
Absorption correction: multi-scan (*SADABS*; Bruker, 2012)	*k* = −11→11
*T*_min_ = 0.542, *T*_max_ = 0.745	*l* = −27→27
71752 measured reflections	

### (Compound-B) Bis{[tris(hydroxymethyl)methyl]ammonium} decaaquadi-µ4oxido-tetra-µ3-oxido-hexadeca-µ2-oxido-hexaoxidodimanganesedecavanadate dihydrate Refinement

**Table:**

Refinement on *F*^2^	Primary atom site location: structure-invariant direct methods
Least-squares matrix: full	Secondary atom site location: difference Fourier map
*R*[*F*^2^ > 2σ(*F*^2^)] = 0.052	Hydrogen site location: mixed
*wR*(*F*^2^) = 0.114	H atoms treated by a mixture of independent and constrained refinement
*S* = 1.12	*w* = 1/[σ^2^(*F*_o_^2^) + (0.0062*P*)^2^ + 110.7865*P*] where *P* = (*F*_o_^2^ + 2*F*_c_^2^)/3
3936 reflections	(Δ/σ)_max_ < 0.001
385 parameters	Δρ_max_ = 0.78 e Å^−^^3^
6 restraints	Δρ_min_ = −1.11 e Å^−^^3^

### (Compound-B) Bis{[tris(hydroxymethyl)methyl]ammonium} decaaquadi-µ4oxido-tetra-µ3-oxido-hexadeca-µ2-oxido-hexaoxidodimanganesedecavanadate dihydrate Special details

**Table:**

Geometry. All e.s.d.'s (except the e.s.d. in the dihedral angle between two l.s. planes) are estimated using the full covariance matrix. The cell e.s.d.'s are taken into account individually in the estimation of e.s.d.'s in distances, angles and torsion angles; correlations between e.s.d.'s in cell parameters are only used when they are defined by crystal symmetry. An approximate (isotropic) treatment of cell e.s.d.'s is used for estimating e.s.d.'s involving l.s. planes.

### (Compound-B) Bis{[tris(hydroxymethyl)methyl]ammonium} decaaquadi-µ4oxido-tetra-µ3-oxido-hexadeca-µ2-oxido-hexaoxidodimanganesedecavanadate dihydrate Fractional atomic coordinates and isotropic or equivalent isotropic displacement parameters (Å^2^)

**Table:**

	*x*	*y*	*z*	*U*_iso_*/*U*_eq_	Occ. (<1)
Mn1	0.80093 (5)	0.43409 (10)	0.18078 (4)	0.0239 (2)	
V2	0.60396 (5)	0.43006 (10)	0.17920 (4)	0.0172 (2)	
V3	0.62652 (5)	0.43454 (11)	0.31542 (4)	0.0182 (2)	
V4	0.47896 (5)	0.58914 (11)	0.11421 (5)	0.0226 (3)	
V5	0.47615 (5)	0.27690 (11)	0.11376 (5)	0.0222 (2)	
V6	0.5000	0.59971 (13)	0.2500	0.0165 (3)	
V7	0.5000	0.26656 (14)	0.2500	0.0153 (3)	
O1	0.6885 (2)	0.4257 (4)	0.17944 (18)	0.0228 (9)	
O2	0.7094 (2)	0.4358 (5)	0.31283 (18)	0.0239 (9)	
O3	0.5769 (2)	0.5689 (4)	0.12912 (18)	0.0223 (9)	
O4	0.5757 (2)	0.2948 (4)	0.12929 (18)	0.0195 (9)	
O5	0.59743 (19)	0.5594 (4)	0.24810 (17)	0.0185 (8)	
O6	0.59724 (18)	0.3057 (4)	0.24851 (17)	0.0148 (8)	
O7	0.6131 (2)	0.5718 (4)	0.36695 (18)	0.0234 (9)	
O8	0.6144 (2)	0.2967 (4)	0.36707 (18)	0.0220 (9)	
O9	0.4718 (3)	0.7085 (5)	0.0667 (2)	0.0362 (12)	
O10	0.4714 (2)	0.4332 (5)	0.06979 (18)	0.0255 (9)	
O11	0.4680 (2)	0.1569 (5)	0.0664 (2)	0.0324 (11)	
O12	0.4913 (2)	0.7043 (4)	0.19037 (19)	0.0251 (10)	
O13	0.49147 (19)	0.4337 (4)	0.19135 (17)	0.0157 (8)	
O14	0.4907 (2)	0.1636 (4)	0.18997 (18)	0.0186 (9)	
O1W	0.9115 (3)	0.4350 (7)	0.1854 (4)	0.056 (2)	
O2W	0.8106 (3)	0.2701 (6)	0.2493 (2)	0.0282 (12)	
O3W	0.7972 (3)	0.2741 (5)	0.1120 (2)	0.0260 (10)	
O4W	0.8094 (3)	0.5982 (5)	0.2475 (2)	0.0255 (10)	
O5W	0.7904 (4)	0.5909 (8)	0.1149 (3)	0.0463 (17)	
H1A	0.929 (6)	0.497 (12)	0.182 (5)	0.08 (4)*	
H1B	0.936 (4)	0.383 (8)	0.187 (3)	0.015 (19)*	
H2A	0.832 (4)	0.227 (8)	0.248 (3)	0.01 (2)*	
H2B	0.820 (5)	0.297 (10)	0.286 (4)	0.06 (3)*	
H3A	0.820 (5)	0.220 (10)	0.119 (4)	0.05 (3)*	
H3B	0.799 (4)	0.295 (8)	0.075 (4)	0.03 (2)*	
H4A	0.841 (6)	0.653 (11)	0.245 (4)	0.08 (4)*	
H4B	0.775 (5)	0.636 (9)	0.245 (4)	0.04 (3)*	
H5A	0.812 (6)	0.626 (12)	0.119 (5)	0.06 (5)*	
H5B	0.774 (7)	0.564 (14)	0.082 (6)	0.11 (5)*	
N1	0.8230 (3)	0.5979 (5)	0.3845 (2)	0.0246 (12)	
H1C	0.7881	0.6509	0.3932	0.029*	
H1D	0.8090	0.5474	0.3529	0.029*	
H1E	0.8588	0.6500	0.3770	0.029*	
C10	0.8451 (3)	0.5061 (7)	0.4357 (3)	0.0275 (14)	
C11	0.8775 (4)	0.5944 (7)	0.4861 (3)	0.0341 (16)	
H11B	0.8885	0.5386	0.5211	0.041*	
H11C	0.9205	0.6343	0.4758	0.041*	
O11A	0.8302 (3)	0.7007 (6)	0.4981 (3)	0.0517 (15)	
H11A	0.8462	0.7751	0.4899	0.078*	
C12	0.8985 (4)	0.4081 (8)	0.4159 (4)	0.047 (2)	
H12B	0.9374	0.4588	0.4034	0.056*	
H12C	0.9159	0.3489	0.4484	0.056*	
O12A	0.8665 (4)	0.3279 (6)	0.3681 (3)	0.0591 (18)	
H12A	0.8860	0.2534	0.3679	0.089*	
C13	0.7796 (5)	0.4328 (9)	0.4506 (4)	0.050 (2)	
H13C	0.7442	0.4997	0.4573	0.061*	0.694 (13)
H13D	0.7615	0.3755	0.4177	0.061*	0.694 (13)
H13E	0.7442	0.4383	0.4170	0.061*	0.306 (13)
H13F	0.7902	0.3369	0.4580	0.061*	0.306 (13)
O13A	0.7939 (7)	0.3553 (10)	0.4991 (4)	0.073 (4)	0.694 (13)
H13A	0.8064	0.2791	0.4896	0.110*	0.694 (13)
O13B	0.7539 (9)	0.487 (3)	0.4988 (9)	0.062 (8)	0.306 (13)
H13B	0.73 (2)	0.44 (3)	0.510 (12)	0.093*	0.306 (13)
O6WA	0.6367 (5)	0.4696 (11)	0.0226 (4)	0.051 (3)	0.694 (13)
H6A	0.617 (6)	0.465 (16)	0.053 (3)	0.077*	0.694 (13)
H6B	0.612 (6)	0.509 (16)	−0.003 (4)	0.077*	0.694 (13)
O6WB	0.6316 (9)	0.596 (3)	0.0209 (8)	0.046 (6)	0.306 (13)
H6C	0.627 (13)	0.58 (4)	0.055 (4)	0.069*	0.306 (13)
H6D	0.595 (8)	0.58 (3)	0.000 (8)	0.069*	0.306 (13)

### (Compound-B) Bis{[tris(hydroxymethyl)methyl]ammonium} decaaquadi-µ4oxido-tetra-µ3-oxido-hexadeca-µ2-oxido-hexaoxidodimanganesedecavanadate dihydrate Atomic displacement parameters (Å^2^)

**Table:**

	*U*^11^	*U*^22^	*U*^33^	*U*^12^	*U*^13^	*U*^23^
Mn1	0.0168 (4)	0.0170 (5)	0.0383 (6)	−0.0012 (4)	0.0049 (4)	−0.0002 (4)
V2	0.0119 (4)	0.0152 (5)	0.0248 (5)	0.0002 (4)	0.0037 (4)	0.0007 (4)
V3	0.0119 (4)	0.0167 (5)	0.0258 (5)	−0.0004 (4)	0.0012 (4)	−0.0009 (4)
V4	0.0206 (5)	0.0193 (6)	0.0279 (6)	0.0007 (4)	0.0024 (4)	0.0071 (4)
V5	0.0191 (5)	0.0225 (6)	0.0252 (6)	−0.0010 (4)	0.0029 (4)	−0.0040 (4)
V6	0.0137 (7)	0.0058 (6)	0.0301 (8)	0.000	0.0025 (6)	0.000
V7	0.0103 (6)	0.0120 (7)	0.0240 (7)	0.000	0.0029 (5)	0.000
O1	0.0148 (19)	0.022 (2)	0.032 (2)	0.0009 (18)	0.0057 (17)	0.0007 (19)
O2	0.0143 (19)	0.027 (2)	0.030 (2)	0.0033 (19)	0.0016 (17)	−0.0001 (19)
O3	0.020 (2)	0.021 (2)	0.027 (2)	−0.0017 (19)	0.0046 (17)	0.0027 (19)
O4	0.017 (2)	0.016 (2)	0.026 (2)	0.0000 (17)	0.0064 (17)	−0.0027 (18)
O5	0.0172 (19)	0.014 (2)	0.024 (2)	0.0007 (17)	0.0026 (16)	−0.0001 (17)
O6	0.0073 (17)	0.0087 (19)	0.029 (2)	0.0015 (15)	0.0031 (15)	0.0002 (16)
O7	0.020 (2)	0.021 (2)	0.028 (2)	−0.0034 (19)	0.0000 (17)	−0.0054 (19)
O8	0.016 (2)	0.020 (2)	0.029 (2)	0.0006 (17)	0.0030 (17)	0.0019 (18)
O9	0.035 (3)	0.032 (3)	0.041 (3)	0.000 (2)	0.002 (2)	0.019 (2)
O10	0.022 (2)	0.031 (2)	0.023 (2)	0.000 (2)	0.0011 (17)	0.005 (2)
O11	0.027 (2)	0.036 (3)	0.034 (3)	−0.004 (2)	0.002 (2)	−0.009 (2)
O12	0.022 (2)	0.019 (2)	0.034 (3)	0.0004 (19)	0.0054 (18)	0.0065 (19)
O13	0.0134 (18)	0.0105 (18)	0.023 (2)	0.0000 (17)	0.0030 (15)	0.0035 (16)
O14	0.0148 (19)	0.0076 (18)	0.034 (2)	−0.0003 (16)	0.0046 (17)	−0.0011 (17)
O1W	0.016 (3)	0.014 (3)	0.139 (7)	0.002 (3)	0.013 (3)	0.002 (3)
O2W	0.031 (3)	0.021 (3)	0.033 (3)	0.010 (2)	0.005 (2)	0.000 (2)
O3W	0.026 (3)	0.017 (2)	0.035 (3)	0.002 (2)	0.004 (2)	0.001 (2)
O4W	0.015 (2)	0.018 (2)	0.045 (3)	−0.003 (2)	0.007 (2)	−0.004 (2)
O5W	0.055 (4)	0.038 (4)	0.046 (4)	−0.014 (3)	0.003 (3)	0.007 (3)
N1	0.024 (3)	0.015 (3)	0.035 (3)	−0.004 (2)	0.002 (2)	−0.001 (2)
C10	0.032 (4)	0.020 (3)	0.030 (3)	0.001 (3)	0.001 (3)	0.002 (3)
C11	0.037 (4)	0.034 (4)	0.031 (4)	−0.002 (3)	0.003 (3)	−0.004 (3)
O11A	0.082 (4)	0.033 (3)	0.039 (3)	0.010 (3)	0.002 (3)	−0.013 (3)
C12	0.049 (5)	0.041 (5)	0.046 (5)	0.018 (4)	−0.012 (4)	−0.008 (4)
O12A	0.082 (5)	0.043 (3)	0.046 (3)	0.038 (3)	−0.020 (3)	−0.021 (3)
C13	0.069 (6)	0.040 (5)	0.045 (5)	−0.030 (5)	0.020 (4)	−0.005 (4)
O13A	0.122 (10)	0.053 (6)	0.041 (5)	−0.038 (7)	−0.005 (5)	0.013 (4)
O13B	0.010 (8)	0.13 (2)	0.049 (11)	−0.012 (10)	0.010 (7)	−0.020 (13)
O6WA	0.059 (6)	0.061 (7)	0.035 (5)	0.007 (5)	0.009 (4)	0.002 (4)
O6WB	0.024 (9)	0.081 (18)	0.034 (10)	−0.008 (10)	0.007 (7)	0.001 (10)

### (Compound-B) Bis{[tris(hydroxymethyl)methyl]ammonium} decaaquadi-µ4oxido-tetra-µ3-oxido-hexadeca-µ2-oxido-hexaoxidodimanganesedecavanadate dihydrate Geometric parameters (Å, º)

**Table:**

Mn1—O1W	2.126 (5)	V7—O6	1.921 (4)
Mn1—O5W	2.139 (7)	V7—O6^i^	1.921 (4)
Mn1—O1	2.169 (4)	V7—O13	2.106 (4)
Mn1—O4W	2.204 (5)	V7—O13^i^	2.106 (4)
Mn1—O3W	2.209 (5)	V7—V5^i^	3.0900 (11)
Mn1—O2W	2.231 (5)	O7—V4^i^	1.884 (4)
V2—O1	1.634 (4)	O8—V5^i^	1.860 (4)
V2—O4	1.789 (4)	O13—V3^i^	2.267 (4)
V2—O3	1.812 (4)	O1W—H1A	0.70 (12)
V2—O6	2.009 (4)	O1W—H1B	0.70 (7)
V2—O5	2.031 (4)	O2W—H2A	0.60 (7)
V2—O13	2.221 (4)	O2W—H2B	0.87 (10)
V2—V3	3.0875 (14)	O3W—H3A	0.70 (9)
V2—V4	3.1056 (14)	O3W—H3B	0.88 (8)
V3—O2	1.609 (4)	O4W—H4A	0.82 (11)
V3—O7	1.821 (4)	O4W—H4B	0.76 (9)
V3—O8	1.821 (4)	O5W—H5A	0.55 (11)
V3—O5	1.992 (4)	O5W—H5B	0.83 (14)
V3—O6	2.010 (4)	N1—C10	1.496 (8)
V3—O13^i^	2.267 (4)	N1—H1C	0.8900
V3—V5^i^	3.1025 (14)	N1—H1D	0.8900
V4—O9	1.587 (5)	N1—H1E	0.8900
V4—O10	1.826 (5)	C10—C12	1.513 (10)
V4—O7^i^	1.884 (4)	C10—C11	1.517 (9)
V4—O3	1.895 (4)	C10—C13	1.525 (10)
V4—O12	2.061 (5)	C11—O11A	1.431 (9)
V4—O13	2.316 (4)	C11—H11B	0.9700
V4—V5	3.0521 (15)	C11—H11C	0.9700
V4—V6	3.0786 (11)	O11A—H11A	0.8200
V5—O11	1.590 (5)	C12—O12A	1.426 (9)
V5—O10	1.824 (5)	C12—H12B	0.9700
V5—O8^i^	1.860 (4)	C12—H12C	0.9700
V5—O4	1.925 (4)	O12A—H12A	0.8200
V5—O14	2.053 (4)	C13—O13A	1.343 (12)
V5—O13	2.334 (4)	C13—O13B	1.36 (2)
V5—V7	3.0900 (11)	C13—H13C	0.9700
V5—V3^i^	3.1025 (14)	C13—H13D	0.9700
V6—O12	1.694 (4)	C13—H13E	0.9700
V6—O12^i^	1.694 (4)	C13—H13F	0.9700
V6—O5	1.928 (4)	O13A—H13A	0.8200
V6—O5^i^	1.928 (4)	O13B—H13B	0.8 (4)
V6—O13^i^	2.097 (4)	O6WA—H6A	0.82 (2)
V6—O13	2.097 (4)	O6WA—H6B	0.82 (2)
V6—V4^i^	3.0786 (11)	O6WB—H6C	0.82 (2)
V7—O14	1.692 (4)	O6WB—H6D	0.82 (2)
V7—O14^i^	1.692 (4)		
			
O1W—Mn1—O5W	92.8 (3)	O12—V6—O12^i^	105.8 (3)
O1W—Mn1—O1	177.2 (2)	O12—V6—O5	96.61 (19)
O5W—Mn1—O1	90.0 (3)	O12^i^—V6—O5	97.57 (19)
O1W—Mn1—O4W	88.0 (2)	O12—V6—O5^i^	97.57 (19)
O5W—Mn1—O4W	87.5 (3)	O12^i^—V6—O5^i^	96.61 (19)
O1—Mn1—O4W	91.99 (18)	O5—V6—O5^i^	156.4 (3)
O1W—Mn1—O3W	89.5 (2)	O12—V6—O13^i^	166.4 (2)
O5W—Mn1—O3W	90.9 (3)	O12^i^—V6—O13^i^	87.80 (18)
O1—Mn1—O3W	90.57 (17)	O5—V6—O13^i^	81.29 (16)
O4W—Mn1—O3W	177.02 (19)	O5^i^—V6—O13^i^	80.50 (16)
O1W—Mn1—O2W	87.9 (3)	O12—V6—O13	87.80 (18)
O5W—Mn1—O2W	179.3 (3)	O12^i^—V6—O13	166.4 (2)
O1—Mn1—O2W	89.35 (19)	O5—V6—O13	80.50 (16)
O4W—Mn1—O2W	92.61 (19)	O5^i^—V6—O13	81.28 (16)
O3W—Mn1—O2W	88.9 (2)	O13^i^—V6—O13	78.6 (2)
O1—V2—O4	102.5 (2)	O12—V6—V4	39.04 (15)
O1—V2—O3	103.9 (2)	O12^i^—V6—V4	144.81 (16)
O4—V2—O3	96.10 (18)	O5—V6—V4	89.45 (12)
O1—V2—O6	97.69 (18)	O5^i^—V6—V4	89.76 (12)
O4—V2—O6	90.65 (17)	O13^i^—V6—V4	127.39 (11)
O3—V2—O6	155.37 (17)	O13—V6—V4	48.76 (10)
O1—V2—O5	99.30 (19)	O12—V6—V4^i^	144.81 (16)
O4—V2—O5	155.64 (17)	O12^i^—V6—V4^i^	39.04 (15)
O3—V2—O5	89.03 (18)	O5—V6—V4^i^	89.76 (12)
O6—V2—O5	75.72 (15)	O5^i^—V6—V4^i^	89.45 (12)
O1—V2—O13	172.67 (19)	O13^i^—V6—V4^i^	48.76 (10)
O4—V2—O13	81.85 (16)	O13—V6—V4^i^	127.39 (11)
O3—V2—O13	81.31 (16)	V4—V6—V4^i^	176.15 (6)
O6—V2—O13	76.23 (14)	O14—V7—O14^i^	107.0 (3)
O5—V2—O13	75.39 (15)	O14—V7—O6	96.90 (17)
O1—V2—V3	88.16 (15)	O14^i^—V7—O6	96.68 (17)
O4—V2—V3	130.47 (14)	O14—V7—O6^i^	96.68 (17)
O3—V2—V3	128.43 (14)	O14^i^—V7—O6^i^	96.90 (17)
O6—V2—V3	39.82 (11)	O6—V7—O6^i^	157.1 (2)
O5—V2—V3	39.40 (12)	O14—V7—O13	87.36 (17)
O13—V2—V3	84.54 (10)	O14^i^—V7—O13	165.61 (18)
O1—V2—V4	137.61 (16)	O6—V7—O13	80.91 (15)
O4—V2—V4	84.31 (13)	O6^i^—V7—O13	81.34 (15)
O3—V2—V4	33.93 (13)	O14—V7—O13^i^	165.61 (18)
O6—V2—V4	124.28 (11)	O14^i^—V7—O13^i^	87.36 (17)
O5—V2—V4	86.88 (11)	O6—V7—O13^i^	81.34 (15)
O13—V2—V4	48.09 (10)	O6^i^—V7—O13^i^	80.91 (15)
V3—V2—V4	119.20 (4)	O13—V7—O13^i^	78.3 (2)
O2—V3—O7	103.4 (2)	O14—V7—V5	38.37 (13)
O2—V3—O8	103.3 (2)	O14^i^—V7—V5	145.37 (15)
O7—V3—O8	95.17 (19)	O6—V7—V5	90.71 (12)
O2—V3—O5	99.42 (19)	O6^i^—V7—V5	88.54 (12)
O7—V3—O5	89.87 (18)	O13—V7—V5	49.01 (11)
O8—V3—O5	154.91 (18)	O13^i^—V7—V5	127.24 (12)
O2—V3—O6	100.03 (19)	O14—V7—V5^i^	145.37 (15)
O7—V3—O6	154.59 (17)	O14^i^—V7—V5^i^	38.37 (13)
O8—V3—O6	88.95 (18)	O6—V7—V5^i^	88.54 (12)
O5—V3—O6	76.57 (15)	O6^i^—V7—V5^i^	90.71 (12)
O2—V3—O13^i^	174.03 (19)	O13—V7—V5^i^	127.24 (12)
O7—V3—O13^i^	80.37 (16)	O13^i^—V7—V5^i^	49.01 (11)
O8—V3—O13^i^	80.84 (16)	V5—V7—V5^i^	176.25 (7)
O5—V3—O13^i^	75.79 (15)	V2—O1—Mn1	176.3 (3)
O6—V3—O13^i^	75.54 (14)	V2—O3—V4	113.8 (2)
O2—V3—V2	89.59 (15)	V2—O4—V5	114.3 (2)
O7—V3—V2	130.18 (15)	V6—O5—V3	107.48 (19)
O8—V3—V2	128.75 (14)	V6—O5—V2	106.83 (18)
O5—V3—V2	40.33 (12)	V3—O5—V2	100.27 (18)
O6—V3—V2	39.80 (11)	V7—O6—V2	106.44 (18)
O13^i^—V3—V2	84.45 (10)	V7—O6—V3	107.72 (18)
O2—V3—V5^i^	136.00 (16)	V2—O6—V3	100.38 (17)
O7—V3—V5^i^	83.42 (14)	V3—O7—V4^i^	114.8 (2)
O8—V3—V5^i^	32.94 (13)	V3—O8—V5^i^	114.9 (2)
O5—V3—V5^i^	124.28 (12)	V5—O10—V4	113.5 (2)
O6—V3—V5^i^	86.64 (11)	V6—O12—V4	109.8 (2)
O13^i^—V3—V5^i^	48.52 (10)	V6—O13—V7	101.55 (16)
V2—V3—V5^i^	119.30 (4)	V6—O13—V2	94.78 (15)
O9—V4—O10	103.9 (2)	V7—O13—V2	93.34 (14)
O9—V4—O7^i^	102.1 (2)	V6—O13—V3^i^	92.73 (14)
O10—V4—O7^i^	91.83 (19)	V7—O13—V3^i^	93.04 (14)
O9—V4—O3	102.0 (2)	V2—O13—V3^i^	168.98 (19)
O10—V4—O3	91.66 (19)	V6—O13—V4	88.32 (14)
O7^i^—V4—O3	154.04 (18)	V7—O13—V4	170.1 (2)
O9—V4—O12	99.6 (2)	V2—O13—V4	86.37 (13)
O10—V4—O12	156.56 (19)	V3^i^—O13—V4	85.80 (13)
O7^i^—V4—O12	83.15 (18)	V6—O13—V5	170.2 (2)
O3—V4—O12	83.47 (18)	V7—O13—V5	88.06 (14)
O9—V4—O13	173.7 (2)	V2—O13—V5	86.46 (13)
O10—V4—O13	82.46 (17)	V3^i^—O13—V5	84.79 (13)
O7^i^—V4—O13	77.84 (16)	V4—O13—V5	82.05 (13)
O3—V4—O13	77.14 (16)	V7—O14—V5	110.9 (2)
O12—V4—O13	74.10 (15)	Mn1—O1W—H1A	120 (9)
O9—V4—V5	137.1 (2)	Mn1—O1W—H1B	132 (6)
O10—V4—V5	33.24 (13)	H1A—O1W—H1B	107 (10)
O7^i^—V4—V5	83.91 (14)	Mn1—O2W—H2A	119 (7)
O3—V4—V5	85.01 (14)	Mn1—O2W—H2B	116 (6)
O12—V4—V5	123.32 (13)	H2A—O2W—H2B	101 (9)
O13—V4—V5	49.23 (10)	Mn1—O3W—H3A	115 (8)
O9—V4—V6	130.8 (2)	Mn1—O3W—H3B	121 (5)
O10—V4—V6	125.37 (14)	H3A—O3W—H3B	107 (9)
O7^i^—V4—V6	78.24 (13)	Mn1—O4W—H4A	115 (7)
O3—V4—V6	78.80 (13)	Mn1—O4W—H4B	108 (6)
O12—V4—V6	31.19 (12)	H4A—O4W—H4B	109 (9)
O13—V4—V6	42.92 (10)	Mn1—O5W—H5A	108 (10)
V5—V4—V6	92.13 (4)	Mn1—O5W—H5B	114 (9)
O9—V4—V2	134.20 (18)	H5A—O5W—H5B	126 (10)
O10—V4—V2	81.76 (13)	C10—N1—H1C	109.5
O7^i^—V4—V2	123.37 (13)	C10—N1—H1D	109.5
O3—V4—V2	32.27 (13)	H1C—N1—H1D	109.5
O12—V4—V2	81.98 (12)	C10—N1—H1E	109.5
O13—V4—V2	45.54 (9)	H1C—N1—H1E	109.5
V5—V4—V2	60.90 (3)	H1D—N1—H1E	109.5
V6—V4—V2	61.87 (3)	N1—C10—C12	107.0 (6)
O11—V5—O10	104.4 (2)	N1—C10—C11	107.9 (5)
O11—V5—O8^i^	102.2 (2)	C12—C10—C11	110.4 (6)
O10—V5—O8^i^	92.89 (19)	N1—C10—C13	106.5 (6)
O11—V5—O4	102.3 (2)	C12—C10—C13	112.3 (7)
O10—V5—O4	90.75 (18)	C11—C10—C13	112.4 (6)
O8^i^—V5—O4	153.41 (18)	O11A—C11—C10	109.8 (6)
O11—V5—O14	99.8 (2)	O11A—C11—H11B	109.7
O10—V5—O14	155.59 (19)	C10—C11—H11B	109.7
O8^i^—V5—O14	84.32 (17)	O11A—C11—H11C	109.7
O4—V5—O14	81.60 (17)	C10—C11—H11C	109.7
O11—V5—O13	173.5 (2)	H11B—C11—H11C	108.2
O10—V5—O13	82.00 (17)	C11—O11A—H11A	109.5
O8^i^—V5—O13	78.27 (16)	O12A—C12—C10	108.9 (6)
O4—V5—O13	76.18 (15)	O12A—C12—H12B	109.9
O14—V5—O13	73.68 (14)	C10—C12—H12B	109.9
O11—V5—V4	137.74 (19)	O12A—C12—H12C	109.9
O10—V5—V4	33.29 (14)	C10—C12—H12C	109.9
O8^i^—V5—V4	84.94 (14)	H12B—C12—H12C	108.3
O4—V5—V4	83.76 (13)	C12—O12A—H12A	109.5
O14—V5—V4	122.39 (12)	O13A—C13—C10	110.4 (9)
O13—V5—V4	48.72 (10)	O13B—C13—C10	112.4 (11)
O11—V5—V7	130.60 (19)	O13A—C13—H13C	109.6
O10—V5—V7	124.92 (15)	C10—C13—H13C	109.6
O8^i^—V5—V7	78.82 (13)	O13A—C13—H13D	109.6
O4—V5—V7	77.53 (12)	C10—C13—H13D	109.6
O14—V5—V7	30.77 (11)	H13C—C13—H13D	108.1
O13—V5—V7	42.93 (10)	O13B—C13—H13E	109.1
V4—V5—V7	91.65 (4)	C10—C13—H13E	109.1
O11—V5—V3^i^	134.32 (17)	O13B—C13—H13F	109.1
O10—V5—V3^i^	82.75 (14)	C10—C13—H13F	109.1
O8^i^—V5—V3^i^	32.17 (13)	H13E—C13—H13F	107.8
O4—V5—V3^i^	122.87 (13)	C13—O13A—H13A	109.5
O14—V5—V3^i^	82.10 (11)	C13—O13B—H13B	109.5
O13—V5—V3^i^	46.69 (9)	H6A—O6WA—H6B	110 (4)
V4—V5—V3^i^	60.92 (3)	H6C—O6WB—H6D	110 (4)
V7—V5—V3^i^	61.69 (3)		
			
O1—V2—O3—V4	174.8 (2)	O14—V5—O10—V4	−6.4 (6)
O4—V2—O3—V4	70.3 (2)	O13—V5—O10—V4	−1.5 (2)
O6—V2—O3—V4	−34.8 (6)	V7—V5—O10—V4	−2.1 (3)
O5—V2—O3—V4	−85.9 (2)	V3^i^—V5—O10—V4	45.64 (19)
O13—V2—O3—V4	−10.5 (2)	O9—V4—O10—V5	−178.9 (3)
V3—V2—O3—V4	−86.4 (2)	O7^i^—V4—O10—V5	−76.0 (2)
O9—V4—O3—V2	−176.2 (3)	O3—V4—O10—V5	78.3 (2)
O10—V4—O3—V2	−71.7 (2)	O12—V4—O10—V5	1.0 (6)
O7^i^—V4—O3—V2	25.9 (6)	O13—V4—O10—V5	1.5 (2)
O12—V4—O3—V2	85.3 (2)	V6—V4—O10—V5	0.9 (3)
O13—V4—O3—V2	10.2 (2)	V2—V4—O10—V5	47.49 (19)
V5—V4—O3—V2	−39.1 (2)	O12^i^—V6—O12—V4	−179.3 (3)
V6—V4—O3—V2	54.1 (2)	O5—V6—O12—V4	80.9 (2)
O1—V2—O4—V5	−176.1 (2)	O5^i^—V6—O12—V4	−80.2 (2)
O3—V2—O4—V5	−70.4 (2)	O13^i^—V6—O12—V4	0.7 (9)
O6—V2—O4—V5	85.9 (2)	O13—V6—O12—V4	0.73 (19)
O5—V2—O4—V5	30.8 (6)	V4^i^—V6—O12—V4	179.93 (3)
O13—V2—O4—V5	9.9 (2)	O14^i^—V7—O14—V5	178.4 (3)
V3—V2—O4—V5	85.5 (2)	O6—V7—O14—V5	−82.4 (2)
V4—V2—O4—V5	−38.54 (19)	O6^i^—V7—O14—V5	79.1 (2)
O2—V3—O7—V4^i^	−174.8 (2)	O13—V7—O14—V5	−1.86 (18)
O8—V3—O7—V4^i^	−69.9 (2)	O13^i^—V7—O14—V5	−0.3 (8)
O5—V3—O7—V4^i^	85.5 (2)	V5^i^—V7—O14—V5	−179.85 (2)
O6—V3—O7—V4^i^	28.6 (6)	N1—C10—C11—O11A	54.2 (7)
O13^i^—V3—O7—V4^i^	9.9 (2)	C12—C10—C11—O11A	170.8 (6)
V2—V3—O7—V4^i^	84.1 (3)	C13—C10—C11—O11A	−63.0 (8)
V5^i^—V3—O7—V4^i^	−39.0 (2)	N1—C10—C12—O12A	−62.1 (8)
O2—V3—O8—V5^i^	174.5 (2)	C11—C10—C12—O12A	−179.3 (6)
O7—V3—O8—V5^i^	69.4 (2)	C13—C10—C12—O12A	54.4 (9)
O5—V3—O8—V5^i^	−31.4 (6)	N1—C10—C13—O13A	−175.5 (8)
O6—V3—O8—V5^i^	−85.5 (2)	C12—C10—C13—O13A	67.7 (10)
O13^i^—V3—O8—V5^i^	−9.9 (2)	C11—C10—C13—O13A	−57.5 (10)
V2—V3—O8—V5^i^	−85.1 (2)	N1—C10—C13—O13B	−102.3 (14)
O11—V5—O10—V4	179.6 (2)	C12—C10—C13—O13B	140.9 (14)
O8^i^—V5—O10—V4	76.2 (2)	C11—C10—C13—O13B	15.8 (15)
O4—V5—O10—V4	−77.4 (2)		

Symmetry code: (i) −*x*+1, *y*, −*z*+1/2.

### (Compound-B) Bis{[tris(hydroxymethyl)methyl]ammonium} decaaquadi-µ4oxido-tetra-µ3-oxido-hexadeca-µ2-oxido-hexaoxidodimanganesedecavanadate dihydrate Hydrogen-bond geometry (Å, º)

**Table:**

*D*—H···*A*	*D*—H	H···*A*	*D*···*A*	*D*—H···*A*
O1*W*—H1*A*···O14^ii^	0.70 (12)	2.01 (12)	2.703 (7)	167 (12)
O1*W*—H1*B*···O12^iii^	0.70 (7)	2.04 (8)	2.727 (8)	169 (8)
O2*W*—H2*A*···O5^iv^	0.60 (7)	2.12 (7)	2.716 (7)	172 (9)
O2*W*—H2*B*···O12*A*	0.87 (10)	2.01 (10)	2.858 (8)	164 (8)
O3*W*—H3*A*···O7^iv^	0.70 (9)	1.94 (9)	2.636 (7)	176 (10)
O3*W*—H3*B*···O11*A*^v^	0.88 (8)	1.91 (8)	2.752 (8)	160 (7)
O4*W*—H4*A*···O6^vi^	0.82 (11)	1.90 (11)	2.708 (6)	167 (10)
O4*W*—H4*B*···O2*W*^vi^	0.76 (9)	2.12 (9)	2.871 (7)	169 (9)
O5*W*—H5*A*···O8^vi^	0.55 (11)	2.18 (11)	2.725 (10)	170 (16)
O5*W*—H5*B*···O13*A*^v^	0.83 (14)	2.12 (13)	2.699 (12)	127 (12)
O5*W*—H5*B*···O13*B*^v^	0.83 (14)	1.95 (14)	2.77 (2)	168 (13)
N1—H1*C*···O3*W*^vi^	0.89	2.03	2.898 (7)	164
N1—H1*D*···O2	0.89	2.31	3.032 (7)	138
N1—H1*D*···O4*W*	0.89	2.45	3.105 (7)	130
N1—H1*E*···O4^vi^	0.89	1.91	2.787 (6)	166
C11—H11*C*···O11^vi^	0.97	2.46	3.392 (9)	160
O11*A*—H11*A*···O6*WA*^vi^	0.82	1.96	2.758 (12)	166
O12*A*—H12*A*···O3^iv^	0.82	1.94	2.756 (7)	174
C13—H13*E*···O2	0.97	2.40	3.280 (10)	151
O13*B*—H13*B*···O6*WB*^vii^	0.77	1.92	2.60 (2)	148
O6*WA*—H6*A*···O3	0.82 (2)	2.23 (9)	2.966 (9)	150 (15)
O6*WA*—H6*B*···O10^viii^	0.82 (2)	2.16 (5)	2.952 (10)	165 (17)
O6*WB*—H6*C*···O3	0.82 (2)	2.03 (13)	2.802 (17)	156 (29)
O6*WB*—H6*D*···O10^viii^	0.82 (2)	1.93 (8)	2.720 (19)	161 (24)

Symmetry codes: (ii) *x*+1/2, *y*+1/2, *z*; (iii) *x*+1/2, *y*−1/2, *z*; (iv) −*x*+3/2, *y*−1/2, −*z*+1/2; (v) *x*, −*y*+1, *z*−1/2; (vi) −*x*+3/2, *y*+1/2, −*z*+1/2; (vii) *x*, −*y*+1, *z*+1/2; (viii) −*x*+1, −*y*+1, −*z*.
